# Workplace Verbal Violence Toward Romanian Doctors and Nurses: Prevalence, Contributing Factors, and Psychological Correlates

**DOI:** 10.3390/healthcare13070786

**Published:** 2025-04-01

**Authors:** Roxana Elena Rusu, Bianca Hanganu, Magdalena Iorga, Vasile-Cătălin Rusu, Adorata Elena Coman, Beatrice Gabriela Ioan

**Affiliations:** 1 Doctoral School, “Grigore T. Popa” University of Medicine and Pharmacy Iasi, 700115 Iasi, Romania; roxana-elena.rusu@umfiasi.ro; 2 Radiology Department, Faculty of Medicine, “Grigore T. Popa” University of Medicine and Pharmacy Iasi, 700115 Iasi, Romania; 3 Legal-Medicine Department, Faculty of Medicine, “Grigore T. Popa” University of Medicine and Pharmacy Iasi, 700115 Iasi, Romania; beatrice.ioan@umfiasi.ro; 4 Behavioral Sciences Department, Faculty of Medicine, “Grigore T. Popa” University of Medicine and Pharmacy Iasi, 700115 Iasi, Romania; magdalena.iorga@umfiasi.ro; 5 Faculty of Medicine, “Grigore T. Popa” University of Medicine and Pharmacy Iasi, 700115 Iasi, Romania; catalin2rusu@gmail.com (V.-C.R.); elena.coman@umfiasi.ro (A.E.C.); 6 Second Internal Medicine Clinic, Sf. Spiridon Clinical Emergency Hospital, 700111 Iasi, Romania

**Keywords:** workplace violence, verbal violence, nurses, doctors, Romania, occupational health

## Abstract

**Background/Objectives**: Workplace violence is a critical issue in the healthcare sector, with verbal violence being the most common form. This study is the first of its kind in Romania aiming to analyze the prevalence, characteristics, contributing factors, and psychological correlates of verbal workplace violence on doctors and nurses. **Methods**: A cross-sectional study was conducted using a questionnaire distributed online between February and April 2022 to doctors and nurses across Romania. Data were analyzed using descriptive statistics, the chi-squared test, and multivariate logistic regression to evaluate contributing factors and response patterns. **Results**: Out of 7951 participants, 56% of doctors and 9.2% of nurses reported experiencing verbal violence in the past 12 months (*p* = 0.001). Shift work and night shifts significantly increased the risk of verbal violence for both groups. Women were more vulnerable, with higher exposure among nurses (OR = 1.687; *p* = 0.001) and doctors (OR = 1.940; *p* = 0.001). The main aggressors were patients and patients’ relatives in both groups of participants, while vertical violence was more common among doctors. Formal reporting was low, although active reactions were more common. In terms of psychological correlates, doctors reported greater psychological strain than nurses (*p* = 0.001). **Conclusions**: This study highlights a critical need for system-wide interventions to address verbal violence in the Romanian healthcare system. Measures such as reporting protocols, staff training on conflict management, and organizational support systems are essential to prevent verbal violence and combat its contributing factors. Implementing these strategies could significantly improve the safety and well-being of healthcare professionals.

## 1. Introduction

Workplace violence is a significant global issue, with one in five employees reporting having been a victim of such an incident at work during their professional life. Additionally, over half (58.5%) of individuals who reported experiencing workplace violence and harassment encountered it on multiple occasions [1]. The World Health Organization (WHO) defines workplace violence as “incidents in which staff are abused, threatened, or assaulted in work-related circumstances, including during commuting to or from home, involving a threat to their safety, well-being, or health” [2]. In the healthcare system, this issue has shown an upward trend, with prevalence rates reaching as high as 62% [3,4]. Among the significant challenges that healthcare systems worldwide face are staff shortages and limited access to care [5]. These challenges create a tense work environment in the healthcare sector, where medical staff often become the target of patients’ and their relatives’ frustrations [6].

Among all forms of non-physical workplace violence, verbal violence is the most frequently encountered in the medical sector [4]. However, these incidents are rarely re-ported, mainly because many healthcare professionals believe that their actions will not lead to meaningful outcomes or significant changes. This perception makes verbal violence seem like an inherent and unavoidable part of their work environment [7,8].

The consequences of verbal violence on medical staff are far from negligible. Studies show that repeated exposure to this type of incident can lead to demotivation, anxiety, depression, additional stress caused by the duty to interact with the aggressors, absenteeism, and ultimately, a decline in the quality of medical care [8,9].

Verbal violence in the medical sector is a global issue, and the social, cultural, and economic influences have a significant role. A 2017 study conducted in hospitals in China revealed that 39.2% of nurses had been victims of verbal violence [10]. Similarly, in Turkey, 88.8% of healthcare personnel reported being victims of verbal violence at least once during their professional life [11]. In Australia, more than 70% of doctors experienced verbal or written aggression [12]. These findings are particularly concerning in departments such as psychiatric or emergency units, environments that concentrate multiple risk factors [4]. In Japan, a study involving nurses in psychiatric wards highlighted that over half (62.5%) of the workplace violence incidents were cases of verbal violence [13]. A 2021 meta-analysis emphasized that 77% of healthcare staff (doctors and nurses) working in emergency departments were exposed to verbal violence at work [14]. A similar situation was documented among nurses in a psychiatric hospital in Israel, where 88.1% of nurses reported incidents of verbal violence [15].

### The Romanian Healthcare System and Workplace Violence

The Romanian healthcare system faces the same challenges identified globally, yet these are exacerbated by regional and local contexts. Within the European Union, Romania stands out due to the large number of medical professionals trained in higher-educational institutions. In 2019, Romania ranked fifth in the European Union in terms of the number of medical graduates and third for nursing graduates [16]. However, the number of doctors and nurses relative to Romania’s population consistently remains below the European Union average [17]. A key factor contributing to this situation is the massive and continuous migration of medical personnel, primarily towards Western European countries that offer better working conditions, more attractive salaries, and greater professional opportunities. This phenomenon significantly contributes to the improvement of healthcare systems in the destination countries while intensifying the staff shortage in Romania [18]. At the same time, the funding of Romania’s healthcare system has consistently been below the European Union average, despite a steady increase over the past decade. This, combined with unequal access to medical services, has led to the overburdening of hospitals and the neglect of primary healthcare services [17,19]. All these factors shape a vulnerable healthcare system that concentrates specific risk factors for workplace violence.

Research on workplace violence in Romania’s healthcare system is limited and fragmented, with existing studies focusing on specific professional groups or healthcare settings. Most studies focused on medical personnel working in the capital city, Bucharest. A 2015 study by the Bucharest College of Physicians revealed that 85% of doctors reported experiencing verbal violence, and 10.2% reported being subjected to physical assaults [20]. However, this study examined the experiences of physicians, leaving other healthcare professionals underexplored. A 2018 study involving 207 professionals from medical units and the medico-social sector in Bucharest showed that 59% of participants had been exposed to forms of workplace violence, with doctors reporting a higher incidence (38.9%) compared to nurses (7.8%) [21]. While the data obtained are valuable, the small sample size and the absence of an analysis linking workplace violence to specific work conditions or demographic factors further emphasize the need for additional research in this area. Moreover, a 2014 study involving resident doctors from the Carol Davila University of Medicine and Pharmacy in Bucharest highlighted that approximately half of the 384 participants had suffered aggression from a patient during their medical training [22]. A multinational study conducted between January 2021 and April 2022, focusing on incidents of abuse experienced by intensive care unit nurses from five European countries, including Romania, revealed that 62.5% of participants had been victims of abuse. Among Romanian participants, the prevalence was higher, reaching 70.8% (172 out of 243 nurses), ranking second among the five countries involved in the study [23]. Although this study offers important data, it focuses solely on intensive care nurses, leaving out the experiences of other medical personnel who may also be affected by workplace violence.

The aim of our study is to investigate workplace violence in the Romanian healthcare sector in order to identify and characterize the types of violence, contributing factors, and their psychological correlates, with the objective of providing data that can serve as a foundation for future measures to prevent violence in the medical sector of this country.

## 2. Materials and Methods

### 2.1. Study Design

This research was designed as a quantitative cross-sectional study, using a standardized questionnaire distributed online at the national level, targeting doctors and nurses in Romania.

### 2.2. Study Population and Sample Size Estimation

According to official data, at the end of 2022, the Romanian healthcare system employed 374,988 healthcare professionals. Among them, 61.8% were doctors and nurses, specifically 71,293 doctors and 160,596 nurses (of whom 18,925 had higher education and 141,671 had middle-level education). The age group distribution of this healthcare personnel is relatively similar, with 42.2% of doctors and 52.2% of nurses being over 45 years old [24]. The required sample size was calculated using a sample size calculator (www.calculator.net) with a 95% confidence level, a 3.6% margin of error, and a response distribution of 50%. Given the total mentioned population of nurses and doctors, the minimum required sample sizes were 734 doctors and 738 nurses.

### 2.3. Research Instrument

The instrument used for data collection was the Workplace Violence in the Health Sector Country Case Studies Research Instruments—Survey Questionnaire, developed and validated by International Labor Organization (ILO), International Council of Nurses (ICN), World Health Organization (WHO), and Public Services International (PSI) in 2003 [2]. After obtaining permission from the WHO to use the questionnaire (permission request no. 384763), it was translated into Romanian and then back-translated into English to verify content accuracy. Subsequently, it was reviewed to ensure clarity of the information. To assess the internal reliability of the questionnaire, a Scale Reliability Analysis was performed. The intercorrelation matrix provided an overview of the association between the investigated items, confirming that they were appropriately constructed and did not exhibit excessive similarity. The analysis yielded a Cronbach’s alpha value of 0.707, which exceeds the minimum threshold of 0.700 required for questionnaire validation.

The questionnaire consists of 130 questions, divided into five sections. The first section includes 28 questions regarding the socio-demographic and professional characteristics of the participants and information about their workplace. The next two sections, totaling 94 questions, explore various forms of physical and psychological violence (verbal violence, bullying, racial harassment, and sexual harassment) experienced at the workplace in the past year. Each section includes definitions of the investigated concepts to minimize personal interpretation by participants. Those who experienced any form of workplace violence in the last 12 months were invited to describe the incidents in terms of location, perpetrators, and the circumstances of the event. They were also asked to provide details about the frequency of these incidents, their emotional status, and any actions taken following the episodes of violence. The last two sections consist of 5 questions investigating current workplace policies, working conditions, and 3 questions regarding participants’ opinions on factors contributing to violent incidents. Some of these questions are designed with conditional logic, allowing the participants to skip a certain segment based on their response. Other questions have an open-ended field in order to ensure that the participants could provide alternative answers or details to the predefined ones. In this questionnaire, verbal violence is defined as behavior that humiliates, degrades, or demonstrates a lack of respect for a person’s dignity and worth [2].

### 2.4. Data Collection

The questionnaire was created in Google Forms and distributed online to all doctors and nurses in Romania through the Romanian College of Physicians and the Order of General Nurses, Midwives, and Nurses of Romania. The participation link was also shared through a Facebook group dedicated to Romanian nurses, with approximately 40,000 members, and one for Romanian doctors, with about 50,000 members. The questionnaire was available for two months, from 10 February 2022 to 10 April 2022. The inclusion criteria for this research were based on profession: participants had to be either doctors or nurses.

### 2.5. Statistical Analysis

The collected data were compiled and processed using the Statistical Package for the Social Sciences (IBM SPSS), version 18.0. The results are presented as percentages and frequencies to provide a clear overview of the response distribution. To assess relationships and associations between variables, the chi-squared test of independence was used. The threshold for statistical significance was set at *p*-value of 0.05. Multivariate logistic regression analysis was conducted to evaluate the influence of socio-demographic and professional characteristics of the victims on the risk of experiencing verbal violence at the workplace. The Forward Likelihood Ratio method was applied, meaning the model was built stepwise, with variables added based on the Likelihood Ratio test. The results were reported as Odds Ratios (exp(B)) with corresponding 95% Confidence Intervals (CIs).

### 2.6. Ethical Considerations

The study presented in this article is part of a broader doctoral research aimed at analyzing workplace violence in the healthcare sector in Romania. The doctoral research was approved by the Research Ethics Committee of the Grigore T. Popa University of Medicine and Pharmacy Iași through approval No. 52/21.02.2021.

Before completing the questionnaire, participants were informed about the study’s objectives and methodology and provided their informed consent. Responses were completely anonymous, and data confidentiality was maintained throughout the study.

## 3. Results

### 3.1. Participants

The questionnaire was completed by 7951 participants, of which 7203 were nurses (90.59%) and 748 were doctors (9.41%). Regarding the age group distribution, our sample closely reflected the actual healthcare workforce, with 44.7% of nurses and 52.8% of doctors being over 45 years old. The majority of participants were women (92.7% of nurses and 72.3% of doctors) and married (72.7% of nurses and 59.2% of doctors). Regarding professional experience, 32.9% of nurses and 43.7% of doctors had over 20 years of experience. Most participants (75.2% of nurses and 73.8% of doctors) work in the public sector. Among nurses, the majority work in shifts (62.7%), at night (53.7%), and have direct interaction with patients (82.6%). In contrast, only 29.7% of doctors work in shifts, 43.2% work night shifts, and most (91.2%) have direct patient interaction. The primary workplace is a hospital for 59% of nurses and 60.7% of doctors as shown in Table 1.

### 3.2. Prevalence and Characteristics of Verbal Violence Incidents Against Healthcare Personnel

Among all participants, 9.2% of nurses (*n* = 662) and 56% of doctors (*n* = 419) reported experiencing verbal violence in the workplace over the past 12 months (*p* = 0.001). Verbal violence incidents predominantly took place within medical institutions (94.3% of nurses and 95.7% of doctors) (*p* = 0.637), with significantly fewer incidents happening during the commute to work or at the patient’s or victim’s home. Such events occurred far less frequently in the online environment (0.2% of nurses and 0.7% of doctors). Patients were identified as the primary source of verbal violence in the workplace, being involved in 42.3% of incidents reported by nurses and 25.5% of those reported by doctors (*p* = 0.001). Additionally, patients’ relatives represented a significant category of aggressors, with a higher prevalence among doctors (23.6%) compared to nurses (13.7%). Vertical verbal violence (from superiors) was reported more frequently by doctors (28.9%) than by nurses (17.7%) (*p* = 0.001). At the same time, horizontal verbal violence (from another colleague or staff member) was more frequently directed towards nurses (24.8%) than doctors (20.1%) (Table 2).

### 3.3. Socio-Demographic and Professional Characteristics of Verbal Violence Victims

The statistical analysis of the socio-demographic and professional characteristics of the victims highlights significant differences between doctors and nurses, outlining distinct victim profiles. Significant differences were observed for gender (*p* = 0.001), age (*p* = 0.05), marital status (*p* = 0.044), professional experience (*p* = 0.028), work sector (*p* = 0.001), shift work (*p* = 0.001), and night shifts (*p* = 0.001) (Table 3). Among doctors, victims of verbal violence were predominantly women (76.6%), with a similar distribution between those under and over 45 years old. The majority of them worked in the public sector (74.9%), during the day (50.5%), and had over 20 years of professional experience (41.5%). Among nurses, victims were mainly women (92.6%), predominantly under 45 years old (53.8%), married, working in shifts (76%), and at night (67.8%). They were employed in the public sector (82.5%) and had more than 20 years of professional experience (32.8%). In the case of both professional categories, verbal violence was reported by those with direct contact with the patients during their work (94.6% of nurses and 94.7% of doctors).

In the second stage of the analysis, we evaluated the influence of independent variables on the likelihood of experiencing verbal violence in the workplace. For this purpose, we applied multivariate logistic regression separately for each healthcare professional category. The analysis model included socio-demographic variables (age, gender, and marital status) and professional factors (work experience, shift work, and night shifts). Additionally, we evaluated specific workplace factors, such as the direct interaction with patients, patient age groups, and working with vulnerable patient categories (such as patients with disabilities, mental illnesses, or terminally ill). This approach allowed us to identify significant predictors of exposure to verbal violence for each professional group analyzed (Table 4).

The results of the logistic regression indicated that being female was a significant risk factor for both nurses (OR = 1.687; *p* = 0.001) and doctors (OR = 1.940; *p* = 0.001). Furthermore, working shifts (nurses (OR = 1.823; 95% CI: 1.714–2.946; *p* = 0.006) and doctors (OR = 1.808; 95% CI: 1.305–2.505; *p* = 0.001)) and during the 18:00–07:00 interval (nurses (OR = 1.445; 95% CI: 1.135–1.840; *p* = 0.003) and doctors (OR = 1.553; 95% CI: 1.124–2.146; *p* = 0.008)) was associated with an increased risk of experiencing verbal violence for both professional categories.

Among doctors, direct interaction with patients diagnosed with HIV/AIDS significantly increased the risk of verbal violence (OR = 2.638; 95% CI: 1.727–9.572; *p* = 0.014), the same prospect was determined when working with patients with disabilities (OR = 2.638; 95% CI: 1.727–9.572; *p* = 0.014). Statistical significance (*p* < 0.05) was determined for nurses working with people with mental illness, home care patients, terminally ill patients, mothers and child care, occupational medicine, and school medicine (Table 4).

### 3.4. Immediate (Re)actions of Victims and Psychological Distress

Active direct reactions were more frequently adopted by doctors compared to nurses, with 56.1% of doctors reporting that they asked the aggressor to stop compared to 42.3% of nurses (*p* = 0.001). In terms of passive reactions, no significant differences were observed between the two professional categories (*p* > 0.05). Approximately 20% of participants reported taking no action following the incidents (*p* = 0.958), reflecting either a tendency to avoid conflict or a perception that reporting the incident would be ineffective. Formal reports to institutional management were more frequent among doctors (13.8 of doctors compared to 7.9% of nurses), while nurses preferred to report to their hierarchical superiors (32.2% of nurses compared to 21.5% of doctors), with significant differences in both cases (*p* = 0.001). An important aspect is the use of counseling services or legal action, where statistically significant differences were observed (*p* < 0.05). Counseling (*p* = 0.013) was sought by 4.8% of doctors compared to only 2.1% of nurses, and 3.8% of doctors reported the incident to the police or prosecution, whereas none of the nurses took the same action (Table 5).

The psychological distress experienced by the nurses and doctors in relation to verbal violence incidents range from memories and thoughts to feelings of alertness, hypervigilance, and the perception that daily activities require extra effort. The nurses consistently reported lower levels of psychological distress compared to doctors. For instance, 42.7% of doctors reported being highly disturbed by repeated thoughts or memories of the incident (25.8% “Quite a lot” and 16.9% “Very much”), compared to only 27.5% of nurses (19.6% “Quite a lot” and 7.9% “Very much”) (*p* = 0.001). Similarly, avoidance behaviors were more often mentioned by doctors, with 34.9% reporting a high tendency to avoid related thoughts or discussions, whereas this was reported by only 21.9% of nurses (*p* = 0.001). The feeling of being “super-alert” or “on guard” was also more commonly associated with doctors (42.9%) compared to nurses (27.2%) (*p* = 0.001). Furthermore, 39.6% of doctors reported that work-related activities required extra effort, compared to 23.6% of nurses (*p* = 0.001) (Table 6).

### 3.5. Associations Between Verbal Violence, Workplace Safety Perception, and the Institutional Context

The results of our study highlight significant differences between doctors and nurses regarding their perception of workplace safety and awareness of reporting procedures for workplace violence (Table 7). Regarding the level of concern about workplace violence of the verbal violence victims (*p* = 0.001), 23.4% of doctors and 21.1% of nurses reported being highly concerned, reflecting a shared concern across both professional groups. Moreover, a larger proportion of nurses (14.7%) stated that they were not concerned at all, compared to only 9.8% of doctors (*p* = 0.001). Among victims, 48.8% of nurses reported that their institution has procedures in place for reporting workplace violence incidents, compared to only 25.3% of doctors (*p* = 0.001). This significant difference does not necessarily indicate the absence of such procedures but rather suggests a possible lack of communication or insufficient dissemination of these procedures among medical staff, particularly among doctors.

## 4. Discussion

This study highlights that workplace verbal violence is a problem for both nurses and doctors working in the Romanian healthcare system. The main perpetrators are patients and patients’ relatives, and the multivariate logistic regression showed that healthcare professionals (nurses and doctors) that are female, have professional experience between 11 and 20 years, work in shifts and at night, and are in direct contact with patients are more prone to being victims of workplace violence in the Romanian healthcare system. Workplace verbal violence is associated with psychological distress (such as avoidance behaviors and hypervigilance), more pronounced in the case of doctors that were victims. Furthermore, our findings suggest that concern about workplace violence differs between professional groups, with doctors expressing a higher overall level of concern compared to nurses. This is the first study to analyze verbal workplace violence against doctors and nurses in Romania, exploring the characteristics of victims and incidents using a validated and internationally recognized research instrument specifically designed for this purpose.

Globally, workplace violence is a major issue affecting all healthcare systems. A 2023 study involving respondents from 79 countries highlighted that verbal violence is the most common form of workplace violence in the healthcare sector, reported by 40% of participants [25]. However, a meta-analysis indicated that workplace violence in the healthcare sector is statistically less prevalent in Europe compared to countries in Asia, North America, or Australia. Among the contributing factors, cultural differences are frequently cited—both in terms of under-reporting incidents and in the context of social norms and respect toward healthcare professionals [4]. The data obtained in our study indicate a higher prevalence of verbal violence incidents among doctors (56%) compared to nurses (9.82%). This difference may be influenced by the uneven distribution of the two professional categories in the participant sample (90.6% nurses and 9.4% doctors—approximately 1 doctor for every 9 nurses) and potential reporting bias. However, it is noteworthy that when analyzing the frequency of verbal violence episodes, the values obtained are relatively similar for both groups. Our findings differ from existing literature. Romanian nurses report a lower percentage of verbal violence compared to their counterparts in Turkey (49.3%), Ethiopia (49%), and China (68.9%) [26,27,28]. In contrast, the proportion of doctors affected by verbal violence in Romania is comparable to that reported in other countries: Turkey (84.6%), India (89.9%), Israel (58.7%), and Germany (73%) [26,29,30,31].

Romanians’ trust in the healthcare system is among the lowest in Europe and globally. In 2022, only one in four Romanians expressed trust in the healthcare system, and only 20% believed they had access to high-quality medical care [32]. Patient satisfaction in Romania is largely tied to the relationship with their attending physician, who is perceived as playing a key role in the recovery process [33]. In Romanian medical practice, nurses are primarily responsible for daily patient care activities, such as administering treatments, continuous monitoring, and reporting health status changes. In contrast, doctors are evaluated mainly for their clinical competencies, particularly the speed of diagnosis and the effectiveness of prescribed treatments [23,33]. This differentiation in responsibilities may contribute to the discrepancies in the frequency of reported verbal violence incidents between doctors and nurses, as well as the higher proportion of victims among doctors. The Romanian healthcare workforce has shown a slight upward trend. In 2023, official data indicated that compared to the previous year, the number of doctors increased by 2%, while the number of nurses grew by only 0.7% [34]. This disparity in workforce distribution may further influence the differences in reported verbal violence incidents between the two professional groups. Similarly, a study conducted in Palestine reported that doctors were more exposed to workplace violence compared to nurses, although this result was not statistically significant [35].

Our study revealed that doctors are more frequently exposed to constant verbal violence, whereas nurses experience it more sporadically. Our findings contradict results from other international studies, which suggest that nurses are the most frequent victims of workplace violence, including verbal violence, due to their direct and continuous interaction with patients and their families. By the nature of their duties, nurses are responsible for monitoring patients’ condition and administering treatments, while doctors are more involved in the decision-making and diagnostic processes [36]. Moreover, in many cases, nurses serve as the first point of contact for patients and their families with the healthcare system, a factor that increases their risk of becoming victims of workplace violence [37]. In our study, direct interaction with patients was statistically significantly correlated with the risk of becoming a victim of verbal violence for both doctors and nurses, a result consistent with findings from other studies in the literature [36].

This dynamic in workplace relationships becomes particularly relevant when analyzing the profile of aggressors. Patients are frequently identified as the primary perpetrators of verbal violence incidents [23,38]. Our study shows that although patients remain the main source of verbal violence (42.3% for nurses and 25.5% for doctors), patients’ relatives also represent a significant proportion (13.7% for nurses and 23.6% for doctors). This finding is attributed to the considerable psychological stress that the patients’ relatives may experience, often linked to a poor understanding of medical terminology and complex medical diagnoses [39].

The statistical analysis further refined the patient categories with whom nurses and doctors interact, focusing on age groups and levels of vulnerability. The results were statistically significant for nurses working with newborns, elderly patients, and adults, while this correlation was not observed among doctors. These findings suggest that the patients’ age-related vulnerabilities may contribute to the increased risk of verbal violence, particularly among nurses who provide continuous care for these groups.

In the case of nurses, caring for patients with mental health disorders increases the risk of verbal violence, similar to those caring for terminally ill patients. The occurrence of verbal violence against healthcare personnel by patients with mental health conditions is consistently reported in the literature [40,41,42]. Similarly, when caring for elderly or terminally ill patients, certain conditions increase the risk of verbal violence toward healthcare staff. On one hand, patients may suffer from illnesses such as dementia or Alzheimer’s disease, which are often accompanied by confusional states and difficulties in communicating with them [43]. On the other hand, medications prescribed for underlying conditions can cause side effects that impair consciousness, worsening these confusional states. Moreover, elderly and terminally ill patients may experience depression, further complicating the relationship between them and medical staff [44]. Our study also found that nurses working with mothers and children, and particularly newborns, have an increased risk of becoming victims of verbal violence. This situation may be explained by the emotional tension experienced by new mothers or those in the postpartum period, which can manifest reduced tolerance and unrealistic expectations from healthcare staff [36]. Similarly, a study from China highlighted an increased risk of workplace violence in neonatology departments, attributing it to family members’ concerns for their infants [39].

In our study, working with HIV/AIDS patients was identified as a significant risk factor for verbal violence among doctors. This finding is particularly concerning given that people living with HIV/AIDS in Romania report feeling discriminated against [45]. For HIV/AIDS patients, their relationship with healthcare staff may be strained due to the stigma surrounding the disease. Healthcare professionals working with this group of patients tend to treat them differently out of fear of infection, leading to uncomfortable interactions [46]. In some cases, these individuals may even face significant difficulties in accessing medical services compared to other patient groups due to refusal of care by healthcare staff [47]. Similarly, a study conducted in Constanța, Romania, revealed that 35% of HIV/AIDS patients experienced discrimination from healthcare staff [48]. These findings underline the challenges of working with this patient group, particularly concerning the difficulties in establishing effective communication with healthcare personnel. This may explain the results obtained in our study. Further research is needed to explore these dynamics more comprehensively.

Our study highlighted the presence of internal verbal violence within the Romanian healthcare system, emphasizing its significant impact on both nurses and doctors. Among nurses, 281 incidents (42.4%) were initiated by another employee, a number similar to incidents caused by patients. The high prevalence of internal verbal violence emphasizes that, beyond improving relationships with patients, it is also crucial to strengthen professional relationships within the medical team, which could ultimately have a significant impact. One of the key factors contributing to internal verbal violence among nurses is the perpetuation of generational practices, where senior employees engage in abusive behavior toward younger colleagues. This phenomenon, combined with the formation of groups and the marginalization of victims, fosters a tense work environment [49,50,51]. On the other hand, institutional deficiencies in staffing and resources correlate with increased demands placed on nurses and contribute to incidents of vertical violence within this professional group [52].

Similarly, doctors in our study reported a high number of internal verbal violence incidents, accounting for nearly half of all their reported cases. Particularly concerning is the high rate of vertical violence, where the aggressor is a member of the institution’s management or a department/service head (28.9% compared to 17.7% among nurses). While vertical violence is frequently reported among nurses, such incidents among doctors are less frequently investigated. A study from Greece highlighted a similarly troubling perspective, with 26.7% of doctors and 31.9% of nurses reporting verbal violence from a senior member of the staff, resulting in devastating effects on job satisfaction, trust in colleagues, and victims’ mental health [53]. Among the most frequently cited contributing factors are poor interpersonal relationships [52,54]. In the case of doctors, these tensions may be due to power struggles between different organizational structures and the male-dominated hierarchy in leadership roles [55]. Managers also blame the media for fostering distrust towards hospitals and medical staff, especially through sensationalist news about doctors and patient care. These factors contribute to the strained relationships among healthcare workers and their relationship with the management [28,54]. Tolerance toward workplace violence in the medical sector is associated with under-reporting of incidents when the aggressor is a colleague. A study in Turkey highlighted that violence from colleagues is often sporadic, and when combined with a general tolerance for workplace violence, it leads to such incidents being rarely reported [26]. Similarly, a study on verbal violence in the emergency department in Saudi Arabia revealed that although 86.6% of employees experienced at least one incident of verbal violence in the past six months, the reporting rate—both formal and informal—was significantly lower when the perpetrator was a colleague or staff member [37].

Our study revealed significant differences between the two professional groups when evaluating the immediate reactions and actions taken by victims of verbal violence. Doctors were more proactive in responding to verbal aggression, engaging in actions such as asking the aggressor to stop, reporting the incident to a colleague, or informing friends or family. In contrast, nurses more frequently reported incidents to their direct supervisors rather than the institution’s management, whereas doctors tended to escalate incidents directly to higher administrative levels. However, it is notable that a relatively small percentage of participants—approximately 20% from both groups—took no action following the incident. A similar percentage was observed in a study from Israel, where 14.8% of affected doctors chose to ignore the incident and continue the medical procedure [30]. In contrast, a study evaluating the reactions of Turkish doctors to violent episodes reported that 36% took no action, and 12% contacted the police [56]. Even more striking, a study conducted in a public hospital in Palestine found that more than half of the victims (56.3%) did not report the incident, believing that reporting it would be useless [35].

The lack of response from healthcare staff may be linked to a high level of tolerance toward episodes of violence, perceived as an integral part of their profession [56]. This may also be connected to the absence of formal reports and the limited involvement of the police or other authorities in such cases. Victims may feel that verbal violence incidents are either not severe enough to involve authorities, mainly due to the lack of visible consequences, or they may wish to avoid lengthy legal procedures [27,30]. In some instances, victims may even be advised not to pursue formal actions [27]. Similarly, Turkish doctors have mentioned the lack of time as the main reason for not reporting such incidents. Other concerns mentioned were the fear of conflict escalation and the potential deterioration of the doctor–patient relationship [56].

The univariate analysis revealed statistically significant differences between the socio-demographic and professional profiles of victims of workplace verbal violence, highlighting certain distinct patterns.

Women were significantly more affected by verbal violence, with 92.6% of nurse victims and 76.6% of doctor victims being female. Moreover, this factor remained statistically significant in the multivariate analysis across professional categories. The same gender distribution is observed in Romania’s medical workforce, where 71% of doctors and 90.7% of nurses are female [34]. This trend mirrors the global situation, where women working in healthcare continue to face challenges related to gender-based inequalities, impacting both their career progression and safety at work [57,58]. The relationship between gender and exposure to workplace violence is inconsistently reported in the international literature. While global studies do not consistently identify gender as a significant factor, research conducted in countries like Ethiopia and Australia highlights a correlation between gender and vulnerability to verbal violence [12,27]. An Italian study evaluating violent incident reports in a university hospital over a three-year period reported a higher frequency of verbal violence against female nurses [59]. Among the factors contributing to this gender-based vulnerability, studies frequently mention cultural influences where men are perceived as superior to women and considered better trained [27].

Our study found no statistically significant correlations regarding the location of violent incidents. Although international regulations acknowledge that workplace violence in the healthcare sector extends beyond medical institutions, in our study, the majority of these incidents (over 90%) occurred within healthcare facilities for both professional groups. This finding aligns with studies conducted in Ethiopia and China, where hospitals are identified as the primary setting for healthcare workers facing aggression [27,60]. In Romania, the tendency of patients to bypass primary care and seek treatment directly in hospitals contributes to the overburdening of medical staff and increases the risk of verbal violence in hospital environments [16,17]. A similar trend has been observed in Turkey, where hospital doctors are more exposed to verbal violence than those working in primary care [56]. Regarding the work sector, our study identified the public sector as a risk factor for verbal violence, a finding consistent with data from Turkey and India [61,62].

Regarding the age of victims, our analysis revealed that doctors over the age of 45 are more frequently victims of verbal violence, while nurses under the age of 45 are more exposed. However, the multivariate analysis confirmed age as a significant risk factor only for nurses. These results are inconsistent across the existing literature. In Ethiopia, nurses over the age of 41 are more susceptible to violence, whereas in China, the risk is higher for nurses under 25 years old [27,60]. Similarly, in Australia, general practitioners with limited professional experience are considered more vulnerable to workplace violence due to their lower hierarchical status and lack of conflict management skills [12]. In China, patients prefer physicians with extensive professional experience, leading to a greater work stress for this category [63]. Older nurses might not be as involved in their work and relationship with patients [27]. In the case of younger or less experienced nurses, their limited clinical experience and poor communication skills may translate into a lack of confidence that might be easily recognized by others [52].

The multivariate analysis in our study confirmed that working in shifts and during night hours increases the risk of verbal violence for both doctors and nurses. This finding is supported by previous studies, which also identify shift work as a major risk factor for healthcare personnel, particularly for nurses [28,60]. Even in large hospitals, afternoon and night shifts are often affected by staff shortages, leading to the overburdening of medical staff and increasing their vulnerability to violent behavior [59]. This vulnerability is also linked with the high number of patients requiring medical evaluation and treatment during these hours, which, in turn, exacerbates other factors such as longer waiting times [30]. In addition to the increased workload, patients are often in critical condition and accompanied by family members, which can further increase the risk of violent outbursts [64].

Our data reveal that doctors report greater psychological strain following verbal violence incidents. The evaluated psychological associations include intrusive memories and the avoidance of thoughts and discussion, but also the perception that daily activities require extra effort. One possible explanation for this observation may be linked to patients’ high expectations of doctors. On the other hand, being confident might translate into arrogance, which can further weaken the relationship between patient and doctors [65]. However, this contrasts with findings from a study in Greece, where nurses reported experiencing more severe psychological consequences associated with verbal violence incidents than doctors with effects on mental health and job satisfaction [53]. Healthcare workers who are frequently exposed to workplace violence are at risk of developing post-traumatic stress disorder, chronic stress, and emotional and physical exhaustion [49,66]. Furthermore, internal violence imposes constant pressure that negatively impacts relationships and teamwork, a situation made worse by the absence of institutional support. In such environments, affected healthcare workers tend to avoid conflicts and isolate themselves, which weakens collaboration within medical teams [49]. Victims often downplay the frequency or seriousness of these incidents, attributing them to their own perceived shortcomings or mistakes [49]. A national study found that 36% of Romanian doctors are at high risk of burnout, particularly among younger professionals [67]. This is concerning, as burnout affecting healthcare workers has many consequences, including the negative impact on their relationships with patients and colleagues [68,69]. Another concern among Romanian doctors is the widespread fear of malpractice procedures, which often leads to defensive medical practices and increased stress among doctors [67].

Our results indicate statistically significant differences between doctors and nurses regarding their perception and management of workplace violence. On one hand, victims of verbal violence report a high level of concern, with 23.4% of doctors expressing significant worry compared to 21.1% of nurses. Moreover, doctors (39.4%) more frequently report not being aware of existing reporting procedures and the necessary steps to take within their institutions. Providing adequate support and ensuring the proper documentation of workplace violence incidents are essential for protecting the mental health of healthcare personnel, and greater awareness of these procedures could lead to an increase in incident reporting [70,71]. The data obtained in our study indicate higher awareness levels compared to those reported in the literature. This suggests that while institutional concern about workplace violence exists, further improvements are still necessary to enhance reporting procedures and support systems. For instance, in China, approximately half of nurses were either unaware of reporting procedures or worked in institutions that lacked such procedures, while in public hospitals in Palestine, about 60% of healthcare staff faced similar circumstances [35,60]. The observed differences in our results compared to the literature may be linked to the immediate reactions of victims. Doctors preferred to report incidents to higher management, whereas nurses more often reported incidents to their direct supervisors. A notable development in the fight against workplace violence in Romania is the government decision adopted in October 2023, which mandates employers to clearly inform employees about what constitutes workplace violence, the procedures for reporting incidents, and the protective measures available for victims [72]. This legislative change opens up opportunities for the reassessment of these data in future research, with the aim of evaluating the effectiveness of this legal measure.

### Strengths and Limitations

One of the major strengths of this research is that it is the first study of its kind to investigate workplace verbal violence among doctors and nurses in Romania. This highlights the study’s originality and its significant contribution to understanding this issue within the Romanian healthcare system. In addition, a globally recognized questionnaire was used, ensuring scientific rigor and enabling the comparability of results with other international studies. The use of clear definitions of the analyzed concepts, presented to participants, minimized the risk of subjective interpretations. The distribution of the questionnaire online via Google Forms allowed for the rapid and efficient collection of data, facilitating access to a large number of nurses and doctors from various regions of the country. This aspect, combined with the diversity of participants and nationwide coverage, represents another significant advantage of the study.

However, this study also presents limitations, mainly due to the data collection method. The online distribution of the questionnaire excluded potential participants without internet access or digital skills, particularly those from rural areas. Additionally, the self-selected sample may introduce selection bias, as individuals who chose to participate might be those with more significant experiences of workplace violence. While this possibility cannot be entirely ruled out, the distribution through multiple channels encouraged a broad participation from a diverse group of doctors and nurses. The self-reported responses could involve subjectivity, and the cross-sectional nature of the study does not allow for observing the evolution of the phenomenon over time. Additionally, while the participation link was shared in the main Facebook groups for Romanian healthcare professionals, ensuring that all respondents belonged to the targeted professional categories was not fully possible. However, the use of specialized groups increased the likelihood of reaching the intended audience.

## 5. Conclusions

This study reveals that verbal violence is a widespread issue in Romania’s healthcare system, with 56% of doctors and 9.2% of nurses reporting this type of incident, highlighting the severity of the issue. These findings emphasize the need for targeted institutional policies to address this issue and improve the safety of healthcare personnel. Healthcare institutions should develop structured training programs that focus on conflict management and de-escalation techniques to equip healthcare professionals with effective ways to handle tense situations. The high prevalence of vertical violence reported by doctors, mainly from management, highlights the importance of addressing power dynamics within medical institutions. Healthcare institutions should also foster a culture of zero tolerance for violence by implementing clear disciplinary measures and providing psychological support for affected employees. Additionally, the low formal reporting rate points to the need for creating a safe and supportive environment where healthcare staff feel empowered to report incidents without fear of retaliation. In this case, improving reporting protocols through simplified, confidential, and accessible systems may encourage more victims to document incidents. Our findings can help shape policies aimed at preventing workplace violence and improving the reporting of such incidents within the Romanian healthcare sector. Recent legislative changes in Romania, such as the October 2023 government decision requiring clearer definitions and reporting procedures for workplace violence, represent a positive step. While these regulatory changes represent progress, their effective implementation remains essential to ensuring long-term improvements in workplace safety.

## Figures and Tables

**Table 1:** Socio-demographic and professional characteristics of participants.

Socio-Demographic and Professional Characteristics	Participants (*n* = 7951) *n* (%)	Nurses (*n* = 7203) *n* (%)	Doctors (*n* = 748) *n* (%)
Gender			
Male	735 (9.2)	528 (7.3)	207 (27.7)
Female	7216 (90.8)	6675 (92.7)	541 (72.3)
Age			
Under 45	4338 (54.5)	3985 (55.3)	353 (47.2)
Over 45	3613 (45.5)	3218 (44.7)	395 (52.8)
Marital Status			
Single/Unmarried	1030 (13.0)	879 (12.2)	151 (20.2)
Married	5677 (71.4)	5234 (72.7)	443 (59.2)
Living with a partner	496 (6.2)	415 (5.8)	81 (10.8)
Widowed	151 (1.9)	134 (1.9)	17 (2.3)
Separated/Divorced	597 (7.5)	541 (7.5)	56 (7.5)
Professional Experience			
Less than 5 years	1967 (24.7)	1844 (25.6)	123 (16.4)
6–10 years	1237 (15.6)	1113 (15.5)	124 (16.6)
11–15 years	1042 (13.1)	941 (13.1)	101 (13.5)
16–20 years	1089 (13.7)	1016 (14.1)	73 (9.8)
More than 20 years	2616 (32.9)	2289 (31.8)	327 (43.7)
Employment Sector			
Private sector	1978 (24.9)	1782 (24.7)	196 (26.2)
Public sector	5967 (75.0)	5415 (75.2)	552 (73.8)
Not specified	6 (0.1)	6 (0.1)	-
Work Schedule			
Full-time	7650 (96.2)	6978 (96.9)	672 (89.8)
Part-time	197 (2.5)	152 (2.1)	45 (6.1)
Temporary/Occasional	104 (1.3)	73 (1.0)	31 (4.1)
Shift Work			
No	2965 (37.3)	2439 (33.9)	**526 (70.3)**
Yes	4986 (62.7)	4764 (66.1)	222 (29.7)
Working Between 6:00 p.m. and 7:00 a.m.			
No	3681 (46.3)	3258 (45.2)	423 (56.5)
Yes	4268 (53.7)	3945 (54.8)	323 (43.2)
Not disclosed	2	-	2 (0.3)
Primary Workplace			
Hospital	4707 (59.2)	4253 (59.0%)	454 (60.7)
Other medical institutions including palliative care, hospice, military units, school medical office	3136 (39.4)	2950 (39.7)	280 (37.4)
Not disclosed	108 (1.4)	94 (1.3)	14 (1.9)
Contact with Patients During Work			
No	1385 (17.4)	1319 (18.3%)	66 (8.8%)
Yes	6566 (82.6)	5884 (81.7%)	682 (91.2%)

**Table 2:** Characteristics of verbal violence incidents.

Characteristic	Nurses (*n* = 662) *n* (%)	Doctors (*n* = 419) *n* (%)	*p*-Value *
Frequency of Incidents			**0.003**
Always/Often	90 (13.6)	91 (21.7)	
Sometimes	498 (75.2)	286 (68.3)	
Once	74 (11.2)	42 (10.0)	
Location of the Violent Incident			0.637
Inside the medical institution	624 (94.3)	401 (95.7)	
At the patient’s home	20 (3.0)	5 (1.2)	
Outside the medical institution—on the way to work	6 (0.9)	1 (0.2)	
Outside the medical institution—at participant’s home	6 (0.9)	-	
By phone	3 (0.5)	9 (2.1)	
Online	1 (0.2)	3 (0.7)	
Not specified	2 (0.3)	-	
Source of Verbal Violence			**0.001**
Patient	280 (42.3)	107 (25.5)	
Patient’s relatives	91 (13.7)	99 (23.6)	
Staff member	92 (13.9)	33 (7.9)	
Colleague	72 (10.9)	51 (12.2)	
Institution management member	33 (5.0)	55 (13.1)	
Department/Clinic/Service head	84 (12.7)	66 (15.8)	
A person from the general public	10 (1.5)	8 (1.9)	

* *p*-values for chi-squared test were provided; bold *p*-value indicates statistical significance.

**Table 3:** Socio-demographic and professional characteristics of verbal violence victims.

Socio-Demographic and Professional Characteristics	Nurses (*n* = 662) *n* (%)	Doctors (*n* = 419) *n* (%)	*p*-Value *
Gender			**0.001**
Male	49 (7.4)	98 (23.4)	
Female	613 (92.6)	321 (76.6)	
Age			**0.050**
Under 45	356 (53.8)	209 (49.9)	
Over 45	306 (46.2)	210 (50.1)	
Marital Status			**0.044**
Single/Unmarried	89 (13.4)	89 (21.2)	
Married	451 (68.1)	230 (54.9)	
Living with a partner	45 (6.8)	54 (12.9)	
Widowed	11 (1.7)	9 (2.1)	
Separated/Divorced	66 (10.0)	37 (8.8)	
Professional Experience			**0.028**
Less than 5 years	144 (21.8)	64 (15.3)	
6–10 years	103 (15.6)	77 (18.4)	
11–15 years	103 (15.6)	63 (15.0)	
16–20 years	95 (14.4)	41 (9.8)	
More than 20 years	217 (32.8)	174 (41.5)	
Employment Sector			**0.001**
Private sector	116 (17.5)	105 (25.1)	
Public sector	546 (82.5)	314 (74.9)	
Shift Work			**0.001**
No	159 (24.0)	274 (65.4)	
Yes	503 (76.0)	145 (34.6)	
Working Between 6:00 p.m. and 7:00 a.m.			**0.001**
No	213 (32.2)	211 (50.4)	
Yes	449 (67.8)	207 (49.4)	
Not disclosed	-	1 (0.2)	
Contact with Patients During Work			**0.001**
No	36 (5.4)	22 (5.3)	
Yes	626 (94.6)	397 (94.7)	

* *p*-values for chi-squared test were provided; bold *p*-value indicates statistical significance.

**Table 4:** Multivariate Logistic Regression Models of Risk Factors for Verbal Violence among Nurses and Doctors.

Independent Variable	Nurses			Doctors		
	Odds Ratio	95% CI	*p*-Value *	Odds Ratio	95% CI	*p*-Value *
Gender (Female)	1.687	1.390–2.047	**0.001**	1.940	1.679–2.242	**0.001**
Age Group (<45 years for nurses/≥45 years for doctors)	1.105	1.094–1.117	**0.001**	1.300	0.965–1.750	0.084
Marital Status (Living with partner)	1.211	0.889–1.651	0.225	1.695	1.029–2.790	**0.038**
Professional Experience (11–20 years)	1.640	1.524–2.781	**0.001**	1.139	0.422–2.857	**0.042**
Shift Work (Yes)	1.823	1.714–2.946	**0.006**	1.808	1.305–2.505	**0.001**
Work Between 18:00–07:00 (Yes)	1.445	1.135–1.840	**0.003**	1.553	1.124–2.146	**0.008**
Direct Interaction with Patients (Yes)	1.398	1.099–1.788	**0.006**	2.118	1.371–3.272	**0.017**
Patient Age Group						
Newborns	1.746	1.600–2.925	**0.009**	1.359	0.916–2.017	0.128
Adolescents	0.908	0.725–1.138	0.403	1.375	0.906–2.087	0.135
Adults	1.795	1.661–2.956	**0.015**	0.950	0.595–1.517	0.830
Elderly	1.687	1.594–2.795	**0.001**	1.166	0.830–1.637	0.375
Particular Types of Patients						
Disabilities	1.108	0.921–1.333	0.275	1.890	1.190–3.002	**0.007**
Mental Illnesses	1.623	1.514–2.757	**0.001**	1.407	0.796–2.485	0.240
Home Care Patients	1.424	1.012–2.004	**0.042**	1.038	0.394–2.733	0.940
Terminally Ill Patients	1.751	1.611–2.924	**0.007**	1.272	0.726–2.226	0.272
HIV/AIDS Patients	1.095	0.728–1.648	0.662	2.638	1.727–9.572	**0.014**
Mother and Child Care	1.274	1.057–1.535	**0.011**	1.017	0.671–1.543	0.935
Geriatrics	0.931	0.770–1.126	0.460	0.915	0.575–1.455	0.915
Occupational Medicine	1.907	1.536–2.368	**0.001**	0.602	0.350–1.035	0.066
School Medicine	1.829	1.302–2.569	**0.001**	0.600	0.350–1.035	0.065

* bold *p*-value indicates statistical significance.

**Table 5:** Immediate (Re)Action of the verbal violence victims.

Immediate (Re)Action of the Victims	Nurses (*n* = 662) *n* (%)	Doctors (*n* = 419) *n* (%)	*p*-Value *
Direct Active Reactions			
I told the person to stop	280 (42.3)	235 (56.1)	**0.001**
I told a colleague	146 (22.1)	137 (32.7)	**0.001**
I told friends/family	84 (12.7)	138 (32.9)	**0.001**
I tried to mediate the conflict	5 (0.8)	6 (1.4)	0.219
Passive Reactions			
I took no action	135 (20.4)	86 (20.5)	0.958
I pretended it did not happen	108 (16.3)	57 (13.6)	0.131
Formal Reporting			
I reported it to a senior staff member	33 (5.0)	27 (6.4)	0.188
I reported it to my immediate superior	213 (32.2)	90 (21.5)	**0.001**
I reported it to the management of the medical institution where I work	52 (7.9)	58 (13.8)	**0.001**
I filled out an incident/accident report form	22 (3.3)	6 (1.4)	**0.040**
I sought counseling	14 (2.1)	20 (4.8)	**0.013**
Legal and Organizational Actions			
I sought help from the professional association	1 (0.2)	6 (1.4)	**0.016**
I sought help from the labor union	4 (0.6)	3 (0.7)	0.553
I transferred to another position/workplace	9 (1.4)	20 (4.8)	**0.001**
I requested compensation through legal action	1 (0.2)	5 (1.2)	**0.035**
I reported it to the Police/Prosecutor’s Office and requested criminal prosecution	0 (0)	16 (3.8)	**0.001**

* *p*-values for chi-squared test were provided; bold *p*-value indicates statistical significance.

**Table 6:** Psychological distress reported by the victims.

Psychological Distress **		Not at All	Slightly	Moderately	Quite a Lot	Very Much	*p*-Value *
(a) Repeated memories, thoughts, or images	Nurses (%)	17.2	30.2	25.1	19.6	7.9	**0.001**
	Doctors (%)	9.3	21.2	26.7	25.8	16.9	
(b) Avoidance of thoughts or discussions	Nurses (%)	19.5	31.4	27.2	15.3	6.6	**0.001**
	Doctors (%)	12.2	22.2	30.8	20.8	14.1	
(c) Feeling “super-alert” or “on guard”	Nurses (%)	17.8	24.9	30.1	18.3	8.9	**0.001**
	Doctors (%)	9.1	22.2	25.8	22.7	20.3	
(d) Feeling that activities require extra effort	Nurses (%)	23.3	26	27.2	15.7	7.9	**0.001**
	Doctors (%)	16.9	19.6	23.9	20.5	19.1	

* *p*-values for chi-squared test were provided; bold *p*-value indicates statistical significance. ** Sample sizes: nurses (*n* = 662); doctors (*n* = 419).

**Table 7:** Perceived concern about workplace violence and knowledge of reporting procedures of the victims.

Item	Nurses (*n* = 662) *n* (%)	Doctors (*n* = 419) *n* (%)	*p*-Value *
Concern About Workplace Violence			**0.001**
Not at all	97 (14.7)	41 (9.8)	
Slightly	128 (19.3)	69 (16.5)	
Moderately	188 (28.4)	116 (27.7)	
Quite a lot	109 (16.5)	95 (22.7)	
Very much	140 (21.1)	98 (23.4)	
Reporting Procedures for Workplace Violence Exist in the Institution			**0.001**
No	143 (21.6)	148 (35.3)	
Yes	323 (48.8)	106 (25.3)	
I don’t know	196 (29.6)	165 (39.4)	
Awareness of Reporting Steps for Workplace Violence	*n* = 323	*n* = 106	0.324
No	36 (11.1)	24 (22.6)	
Yes	287 (88.9)	82 (77.4)	

* *p*-values for chi-squared test were provided; bold *p*-value indicates statistical significance.

## Data Availability

The data presented in this study are available on request from the corresponding author. The data are not publicly available due to privacy and ethical issues.

## Abbreviations

The following abbreviations are used in this manuscript:

WHO	World Health Organization
ILO	International Labour Organization
ICN	International Council of Nurses
PSI	Public Services International
SPSS	Statistical Package for the Social Sciences
CI	Confidence Intervals
OR	Odds Ratio
